# Single Point Mutation from E22-to-K in A*β* Initiates Early-Onset Alzheimer's Disease by Binding with Catalase

**DOI:** 10.1155/2020/4981204

**Published:** 2020-12-24

**Authors:** Wenjing Jiang, YanYu, Dandan Yao, Xuechao Fei, Li Ai, Yalan Di, Jingnan Zhang, Xiangpei Yue, Shengjie Zhao, Rongqiao He, Jihui Lyu, Zhiqian Tong

**Affiliations:** ^1^Beijing Institute of Brain Disorders, Laboratory of Brain Disorders, Ministry of Science and Technology, Collaborative Innovation Center for Brain Disorders, Capital Medical University, Beijing, China; ^2^Center for Cognitive Disorders, Beijing Geriatric Hospital, Beijing 100095, China; ^3^Chinese Institute of Rehabilitation Science, China Rehabilitation Research Center, Beijing Key Laboratory of Neural Injury and Rehabilitation, Beijing 100068, China; ^4^Tianjin First Central Hospital, Tianjin 300192, China; ^5^Department of Cognitive Sciences, Institute of Cognition and Brain Sciences, Beijing, China; ^6^State Key Laboratory of Brain & Cognitive Science, Institute of Biophysics, CAS Key Laboratory of Mental Health, University of Chinese Academy of Sciences (UCAS), Beijing 100101, China

## Abstract

Amyloid-beta (A*β*) is a critical etiological factor for late-onset familial Alzheimer's disease (AD). However, an early-onset AD has been found to be related with an A*β* mutation in glutamic acid 22-to-lysine (Italian type E22K). Why only one single point mutation at E22 residue induces AD remains unclear. Here, we report that a Chinese familial AD pedigree with E22K mutation was associated with higher levels of serum hydrogen peroxide (H_2_O_2_) and lower activity of catalase (a H_2_O_2_ degrading enzyme) than controls. Further, we found that E22K binding with catalase caused more severe H_2_O_2_ accumulation in the brains of E22K-injected rats than A*β*-injected rats. Unexpectedly, H_2_O_2_ bound with the mutation site 22K residue of E22K and elicited more rapid aggregation of E22K than A*β in vitro*. Moreover, H_2_O_2_ acted with E22K synergistically to induce higher cellular toxicity than with A*β*. Notably, intrahippocampal infusion of E22K led to more severe plaque deposition, neuron death, and more rapid memory decline than A*β*-injected rats. However, L-cysteine, a H_2_O_2_ scavenger, not only prevented self-aggregation of E22K but also reduced H_2_O_2_-promoted E22K assembly *in vitro*; subsequently, it alleviated Alzheimer-related phenotypes. Hence, E22K binding with catalase promotes the early onset of familial AD, and L-cys may reverse this disease.

## 1. Introduction

Today, the number of people suffering from dementia has reached 50 million, with Alzheimer's disease (AD) accounting for 60% to 70% of all dementia cases [1]. Accumulating evidence indicates that amyloid-beta aggregation of (A*β*) peptide, which is cleaved from amyloid precursor proteins, contributes to the pathogenesis of late-onset AD [2]. The most common variants of A*β* are 40 (A*β*40) and 42 (A*β*42). Although A*β*42 is expressed at a much lower level than A*β*40, it shows higher cell toxicity and has been found to be the initial and major component in cerebral senile plaques (SP) [3]. In most late-onset AD cases, the disease appears to be sporadic, with an average onset age of 65 years. However, several A*β* mutations lead to the emergence of AD symptoms before the age of 65 years, and the resulting disease is known as early-onset familial AD [4].

Mutations clustering near the A*β* N-terminus can alter A*β* production and enhance the kinetics of fibril and intermediate aggregate species formation [5, 6], while mutations located at the A*β* C-terminus are shown to affect the release of A*β* by accelerating its production [7, 8]. Interestingly, the mutations reported within residues 21–23 of A*β* are implicated in not only increasing A*β* production but also enhancing A*β* aggregation kinetics and/or delaying A*β* clearance [9–11]. Notably, in 1999, a rare early-onset dementia in Italian familial AD patients was found to be associated with an A*β* mutation in glutamic acid 22-to-lysine (Italian type E22K) and rapid cognitive decline [12]. A*β*-induced oxidative stress is widely considered to accelerate the process of AD development [13]. Whether E22K induces oxidative stress and self-aggregation remains to be elucidated.

In this study, we investigated the role of E22K in a Chinese familial early-onset AD pedigree and E22K-injected rat model. The results showed that E22K-inactivated catalase caused a marked elevation in hydrogen peroxide (H_2_O_2_) levels. In turn, H_2_O_2_ binding to the K22 residue of E22K triggered earlier and more rapid memory decline in the early-onset AD rat model following injection of E22K compared with the late-onset AD rat model following injection of wild-type A*β*. However, E22K-induced aggregation and memory impairments were reversed following treatment with L-cys.

## 2. Materials and Methods

### 2.1. Isolation of Blood from AD Patients and Healthy Donors

The clinical study was registered at the Chinese Clinical Trial Registry (http://www.chictr.org/cn, UniqueIdentifier: ChiCTR-OOC-14005576). The mean age of the 68 elderly patients with AD and 72 age-matched controls was 70.32 ± 2.89 years old, 7 AD patients with E22K mutation was 51.24 ± 3.17 years old, and cognitive functions were assessed using the Mini-Mental State Examination. The blood from the above participants was collected and tested for serum catalase activity and H_2_O_2_ concentrations. Informed consent was obtained from each participant either directly or indirectly from his or her guardian before participation. Ethical approval for the clinical investigations was obtained from the Clinical Ethics Committee at the Capital Medical University, China. The venous blood was used for enzyme activity analysis and gene sequencing (^#^HiSeq 2500, Illumina, USA).

### 2.2. Animals

The animal protocols were approved by the Biologic Research Ethics Committee at Capital Medical University. Adult male Sprague-Dawley (SD) rats (220 ± 20 g) were obtained from the Experimental Animal Center of Capital Medical University, China. All the animals were housed in a temperature-controlled room under a 12 h light-dark cycle with access to water and food *ad libitum*.

### 2.3. Synthetic Polypeptides

Wild-type A*β*42 peptides and single point mutations including lysine 16-to-proline (K16P), phenylalanine 19-to-proline (F19P), glutamic acid 22-to-proline (E22P), glutamic acid 22-to-proline (E22K), aspartic acid 23-to-proline (D23P), and a double-mutation E22P-D23P were synthesized by the ChinaPeptides Co., Ltd. Shanghai, China. Peptide purity (standard: ~96.06%) was analyzed by high-performance liquid chromatography (Agilent HP1100, Palo Alto, USA). The sequences of amino acids of the synthetic peptides were identified by liquid chromatography-mass spectrometry (API 150EX, Palo Alto, USA). Detection parameters were as follows: NEB: 10.00; CUR: 12.00; IS: +4500 TEM: 0.00; flow rate: 0.2 mL/min; and run time: 1 min.

### 2.4. Intrahippocampal Injection of E22K and A*β* in SD Rats

E22K and A*β*42 peptides (1 *μ*g/*μ*L, Sigma-Aldrich, USA) were prepared as described previously [14]. The five groups of SD rats (*n* = 10–13, per group) under general anesthesia with isoflurane were placed in a stereotaxic apparatus and injected in the dentate gyrus (DG) of the dorsal hippocampus bilaterally (anterior posterior, 3.2 mm; lateral, 2.5 mm; horizontal, 3.5 mm from bregma) with one of the following: (1) vehicle (5 *μ*L normal saline, pH = 7.4, used as control); (2) E22K (5 *μ*L, 1 *μ*g/*μ*L); (3) E22K plus L-cys (E22K: 1 *μ*g/*μ*L, 5 *μ*L; L-cys: 0.16 *μΜ*, 5 *μ*L); (4) A*β*42 (1 *μ*g/*μ*L, 5 *μ*L); (5) A*β*42 plus L-cys (A*β*42: 1 *μ*g/*μ*L, 5 *μ*L; L-cys: 0.16 *μ*M, 5 *μ*L) [14]. On the 23^rd^ day after the above injections, all rats were subsequently used in behavioural (Morris water maze) and biochemical tests.

### 2.5. Analysis of H_2_O_2_ and Catalase Activity in Rats and Humans

H_2_O_2_ levels in the hippocampus of rats and in the blood of human test subjects were measured using a commercially available kit in accordance with the manufacturer's instructions (^#^S0038, Jiancheng Co., Nanjing, China). Catalase activity was measured using a commercially available kit in accordance with the manufacturer's instructions (^#^S0051, Beyotime Biotechnology, Jiangsu, China). Synthetic human A*β*42 or E22K (5 *μ*M) was used to inhibit human catalase activity.

### 2.6. Molecular Simulation of E22K/Catalase and H_2_O_2_/E22K

The three-dimensional (3D) crystal structures of human blood catalase (CAT, PDB ID: 1DGF) and A*β* (PDB ID: 1IYT) were downloaded from the Protein Data Bank, at https://www.ncbi.nlm.nih.gov/Structure/pdb/1DGF and https://www.ncbi.nlm.nih.gov/Structure/pdb/1IYT, respectively. The 3D crystal structure of H_2_O_2_ was downloaded from DRUGBANK (https://www.drugbank.ca/drugs/DB11091). L-cys was obtained free of charge from DRUGBANK (https://www.drugbank.ca/drugs/DB00151). Catalase binding of E22K or of A*β* was carried out using the software Discovery studio 3.0. H_2_O_2_ binding of E22K or of A*β* was simulated using the software AutoDockTools-1.5.6 and the MGLTools software (http://mgltools.scripps.edu/). The hydrogen bonds between H_2_O_2_ and the amino acids were analyzed using the software PyMOL 1.7, which can be freely downloaded (http://sourceforge.net/projects/pymol/).

### 2.7. Identification of the Complex of A*β*/Catalase and E22K/Catalase by Western Blotting

The A*β*42 peptides were prepared as described previously. We incubate A*β* (100 *μ*M)/E22K and catalase (80 *μ*M) for one hour at room temperature. Then, coincubated solutions of A*β*/catalase and E22K/catalase were dissolved in sample buffer (8 M urea, 100 mM tricine, 8% sodium dodecyl sulfate (SDS), 30% glycerol, 0.01% phenol red, and 10% mercaptoethanol). The proteins were electrophoretically separated by 10% sodium dodecyl sulfate-polyacrylamide gel electrophoresis and then transferred to polyvinylidene fluoride (PVDF) membranes (0.2 mM pore size, Bio-Rad). The membranes were blocked in fat-free milk at room temperature for 1 h and washed 3 times with TBST buffer solution for 10 min each time. Next, the primary antibody solution A11 (1 : 1000, OriGene, USA) and anti-catalase (1 : 1000, Abcam, UK) were added and incubated at 4°C overnight. The membrane was washed in the same way. Then, samples were incubated with IRDye®800CW goat-anti-rabbit antibody (1 : 5000, Abcam, UK) at room temperature for 1 h and visualized via chemiluminescence with an infrared laser scanning system (Odyssey LI-COR, USA).

### 2.8. Immunofluorescence Analysis of the Colocalization of A*β*42 and Catalase

To visualize colocalization of aggregated A*β* with catalase, human SH-SY5Y cells were coincubated with E22K/A*β* (5 *μ*M dissolved in medium) for 12 h; the cells were fixed with 4% paraformaldehyde in PBS (pH 7.4) and permeabilized with 0.25% Triton-X in PBS, followed by incubation with a rabbit anti-catalase polyclonal antibody (^#^ab48613, Abcam, USA) and 4G8 (^#^SIG-39200, Covance USA) on a shaker at 4°C overnight. Then, HiLyte Fluor 488-labelled A*β* (^#^60479, Anaspec, USA) and Alexa Fluor 647 goat anti-rabbit antibody (^#^ab150079, Abcam, USA) were used. The colocalization was visualized by using soft WoRx image analysis software (Applied Precision, Issaquah, WA).

### 2.9. A*β* Secondary Structure Detected by Th T Dye

The incubated samples were vortex mixed, and 40 mL aliquots were withdrawn and mixed with 960 mL of 10 mM Thioflavin T (Th T) dye in 10 mM phosphate-buffered saline, pH 6.0. The samples were analyzed in a PerkinElmer (Beaconsfield, UK) LS 50 luminescence spectrometer with an excitation at 437 nm and an emission at 485 nm. Slit widths were set to 5 nm, as described previously [15].

### 2.10. Scanning A*β* Fibrils by TEM

A*β* (1 *μ*M) was incubated with formaldehyde (0, 0.5, 5, and 10 mM, respectively) for 30 min at 37°C to observe A*β* oligomer channel formation. After 48 h, A*β* aggregation was analyzed by TEM. The incubated samples were loaded on a carbon-coated grid for 2 min, stained with 2% (*w*/*v*) uranyl acetate for 1 min, and then dehydrated through a graded water-ethanol series. Samples were visualized and photographed with transmission electron microscopy (TEM) (^#^JEOL 100CX at 60 kV, JEOL Ltd., Japan), as described previously [16].

### 2.11. Scanning [Ca^2+^]i by Laser Confocal Microscopy

Injections of E22K, E22K coincubated with H_2_O_2_, A*β*, and A*β* coincubated with H_2_O_2_ were used to stimulate intracellular influx [Ca^2+^]i stained by Fluo 4 in cultured mouse neuroblastoma N2a cells, as previously described [17, 18].

### 2.12. Measurement of Cytotoxicity Using a CCK-8 Kit

N2a cells were cultured as previously described [19]. The viability of N2a cells was measured with a Cell Counting Kit-8 (CCK-8) kit according to the manufacturer's instruction (^#^E1CK-000208-10, EnoGene, Nanjing, China).

### 2.13. Neuron Loss Stained by H&E

The hematoxylin-eosin (H&E) staining was performed as previously described [20].

### 2.14. Brain SP Quantified by Immunochemistry with 4G8, Th S dye, and DAPI

Brain sections were prepared from rats. Senile plaques (SP) in the brain sections were detected with biotinylated monoclonal 4G8 antibody, as described previously [21]. Double staining of plaques blocking the extracellular space (ECS) was performed with DAPI and 1% Th S and visualized using fluorescence microscopy.

### 2.15. Memory Behaviours Assessed by MWM

Memory-related behaviours of rats were analyzed using the Morris water maze (MWM). After injection with E22K or wild-type A*β*, rats were transferred to the platform, and spatial training and memory retrieval experiments were conducted as previously described [22, 23].

### 2.16. Statistical Analysis

The data of the MWM test were analyzed using repeated measures ANOVA, with days as a within-subjects factor and different treatments as a between-subjects factor. The differences between treatment groups within each day were analyzed using one-way ANOVA, and Fisher's Least Significant Difference (LSD) was used for post hoc comparisons. SPSS 16.0 (SPSS Inc., Chicago, IL, USA) was used for all analyses. For other experiments, statistical significance was determined using the Student *t*-test (for independent or dependent samples, as appropriate) with a *p* < 0.05 (two-tailed) considered as statistically significant. All data are reported as mean ± standard errors.

## 3. Results

### 3.1. Oxidative Stress Was Enhanced in a Chinese Familial AD Pedigree with E22K Mutation

**Figure 1:**
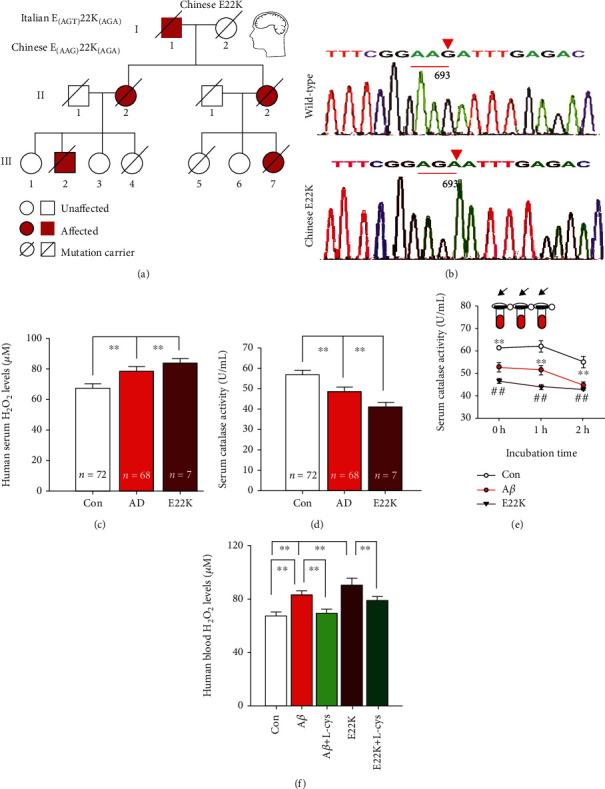
The changes in serum catalase activity and H_2_O_2_ levels in a Chinese familial AD pedigree with E22K mutation. (a) E22K mutation in a Chinese early-onset familial Alzheimer's disease pedigree. (b) APP693 mutation identified by gene sequencing. (c) Human serum H_2_O_2_ levels detected by an H_2_O_2_ kit (Con group: *n* = 72; AD group: *n* = 68, *p* < 0.01). Con: control; E22K: patients with E22K mutant; AD: pure AD patients. (d) Human serum catalase activity analyzed by a catalase kit (*p* < 0.01). (e, f) Changes in human serum catalase activity and H_2_O_2_ levels after E22K and A*β* incubation 0, 1, and 2 h (*n* = 72, *p* < 0.01 vs. control, respectively).

There was a difference in the code-AGT for Glu (E) in Italian E22K mutation [12] and AAG for E in Chinese mutation. We found that a Chinese E22K familial AD pedigree consisted of three generations in which AD was inherited in each generation. The index III2 and III7, who carried the E22K mutation, exhibited the loss of short- and long-term memory, executive function deficits, spatial disorientation, and depression at the age of 48 years (Figure 1(a)). This Chinese E22K mutation (G to A at codon 693, Glu to Lys at residue 22) was identified by gene sequencing (Figure 1(b)).

Oxidative stress is widely considered to accelerate the process of AD development [13]. Thus, we detected H_2_O_2_, a marker of oxidative stress, in the blood isolated from 7 early-onset dementia patients with E22K mutation, 68 AD patients, and 72 age-matched controls. The results showed that the levels of blood H_2_O_2_ in E22K and AD patients were higher than those in controls (83.21 ± 3.76, *n* = 7; 78.32 ± 6.87, *n* = 68; 65.43 ± 5.56, *n* = 72, *p* < 0.01; Figure 1(c)). We then examined serum catalase activity, an H_2_O_2_ degrading enzyme, using a catalase kit. The activity of catalase in AD was obviously lower than that in controls (41.32 ± 1.21, *n* = 7; 48.87 ± 2.65, *n* = 68; 57.88 ± 3.49, *n* = 72, *p* < 0.01; Figure 1(d)).

To mimic the effects of E22K or A*β* on blood catalase activity and H_2_O_2_ levels in early-onset or late-onset AD patients, we incubated the blood from healthy controls with the synthetic E22K (a proposed inhibitor of catalase in this study) and A*β* (a confirmed inhibitor of catalase) [24], respectively (Figure S1). As expected, both E22K and A*β* led to a time-dependent decline in serum catalase activity (*n* = 72, *p* < 0.01); however, E22K incubation exhibited lower activity of catalase than A*β* treatment at the different time points (Figure 1(e)). Furthermore, E22K elicited higher levels of H_2_O_2_ accumulation than A*β* in the blood after a 2 h incubation (86.49 ± 6.17 vs. 81.93 ± 5.34, *n* = 72, *p* < 0.01). Interestingly, L-cysteine (L-cys) can have spontaneous reactions with H_2_O_2_ [25]. As expected, L-cys can decrease H_2_O_2_ accumulation induced by E22K or A*β* in the blood of healthy controls (Figure 1(f)).

### 3.2. E22K Induced Increased H_2_O_2_ Level Compared with A*β* by Inactivating Catalase

**Figure 2:**
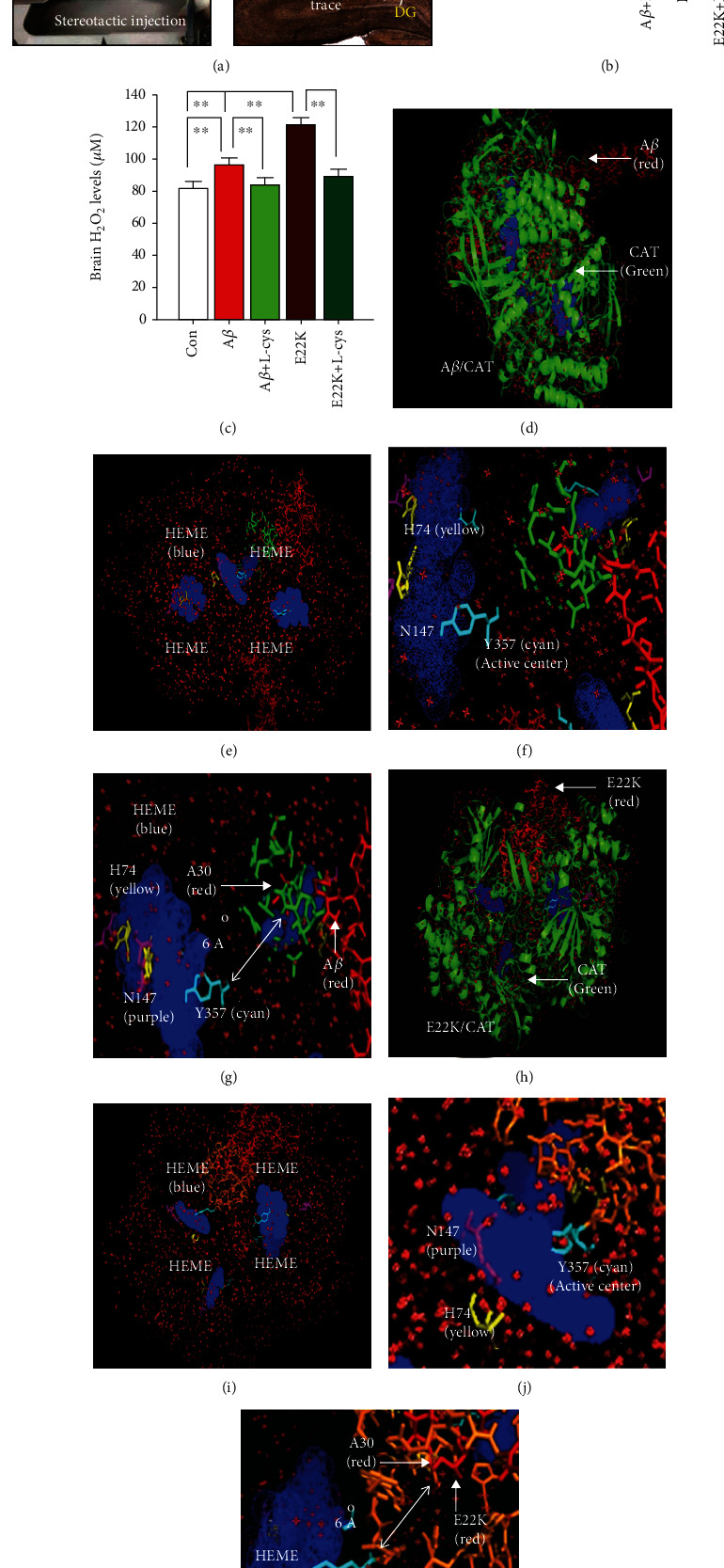
The effects of E22K and A*β* binding with catalase on H_2_O_2_ levels. (a) The schedule of intrahippocampal injection with saline (Con), A*β*, A*β* plus L-cys, E22K, and E22K plus L-cys and a chemical reaction between L-cys and H_2_O_2_ (above). DG: dentate gyrus. The identification of the local injection trace in the brains of SD rats by immunochemistry (below). (b) Brain catalase activity and H_2_O_2_ concentrations in rats analyzed by commercial kits (*n* = 10, *p* < 0.01). (c) Brain catalase activity and H_2_O_2_ concentrations in rats analyzed by commercial kits (*n* = 10, *p* < 0.01). ^∗∗^*p* < 0.01; ^##^*p* < 0.01. Data are the means ± SEM. (d) The simulated three-dimensional (3D) crystal structures of A*β* binding with catalase simulated by Discovery studio 3.0. Catalase (CAT, PDB ID: 1DGF); A*β* (PDB ID: 1IYT). (e) The binding motif, four molecules of HEME (blue), in catalase. (f) Histine 74 (H74, yellow) and asparagine 147 (N147, purple) are the binding sites of H_2_O_2_. Tyrosine 357 (Y357, cyan) is the active center of catalase for Fe(III) oxidation to Fe(IV). (g) The distance between alanine 30 (A30, red) of A*β* and Y357 (cyan) of catalase (over 6 Å). (h–k) The simulated 3D structure of E22K binding with catalase (green), HEME group (blue): four porphyrin heme (iron) groups, H74 (yellow), N47 (purple), and Y357 (cyan). (k) The distance between alanine 30 (A30, red) of E22K and Y357 (cyan) of catalase (less 6 Å).

To test the above notion that E22K increased H_2_O_2_ levels and decreased catalase activity compared with A*β* in animal models, we injected the medicines E22K and A*β* with or without L-cys into the hippocampi of healthy adult male Sprague-Dawley (SD) rats and identified the position of the injection location by immunohistochemistry (Figure 2(a)); we then examined the changes in catalase activity and in H_2_O_2_ levels. Thirty days after either an intrahippocampal injection of E22K plus L-cys, A*β*, or A*β* plus L-cys, E22K-injected rats showed significantly reduced brain catalase activity (45.39 ± 2.36 vs. 56.28 ± 2.04, *n* = 10, *p* < 0.01) and increased H_2_O_2_ levels (119.21 ± 6.47 vs. 88.79 ± 5.17, *n* = 10, *p* < 0.01) compared with A*β*-injected rats (Figures 2(b) and 2(c)). Interestingly, L-cys reversed E22K- and A*β*-induced reductions in catalase activity and increases in H_2_O_2_ levels (Figures 2(b) and 2(c)). These data indicated that E22K-induced elevation in H_2_O_2_ levels is most likely due to inhibition of catalase activity.

### 3.3. E22K Bound More Strongly with Catalase Than Did A*β* In Vitro

Previous studies have shown that A*β* can bind with catalase and induce H_2_O_2_ production [24, 26]. To test whether E22K may bind more frequently with catalase than A*β*, we used molecular simulations to mimic catalase binding with E22K or A*β* by using Discovery studio 3.0. The results showed that catalase theoretically bound with A*β* (Figure 2(d)). Structurally, catalase has four HEME molecules (Figure 2(e)). Histine 74 (H74, yellow) and asparagine 147 (N147, purple) residues in the structure of HEME are known to interact with H_2_O_2_, while tyrosine 357 (Y357, cyan) is the critical active center for Fe(III) oxidation to Fe(IV) (Figures 2(e) and 2(f)). The amino acid with the closest distance between A*β* and HEME Y357 was alanine 30 (A30, red). Notably, the amino acids (green) around A30 6 Å of A*β* did not directly bind with HEME Y357 in catalase (Figure 2(g)), suggesting that the binding between A*β* and catalase was relatively weak. However, E22K bound to catalase more strongly than A*β* (Figures 2(h)–2(k)), because the amino acids (brown) around A30 (red) 6 Å were directly connected with Y357 (cyan) (Figure 2(k)). This simulated result suggested that the mutation from E22 to K22 in familial AD leads to a closer binding between E22K and catalase when compared with A*β* and catalase.

**Figure 3:**
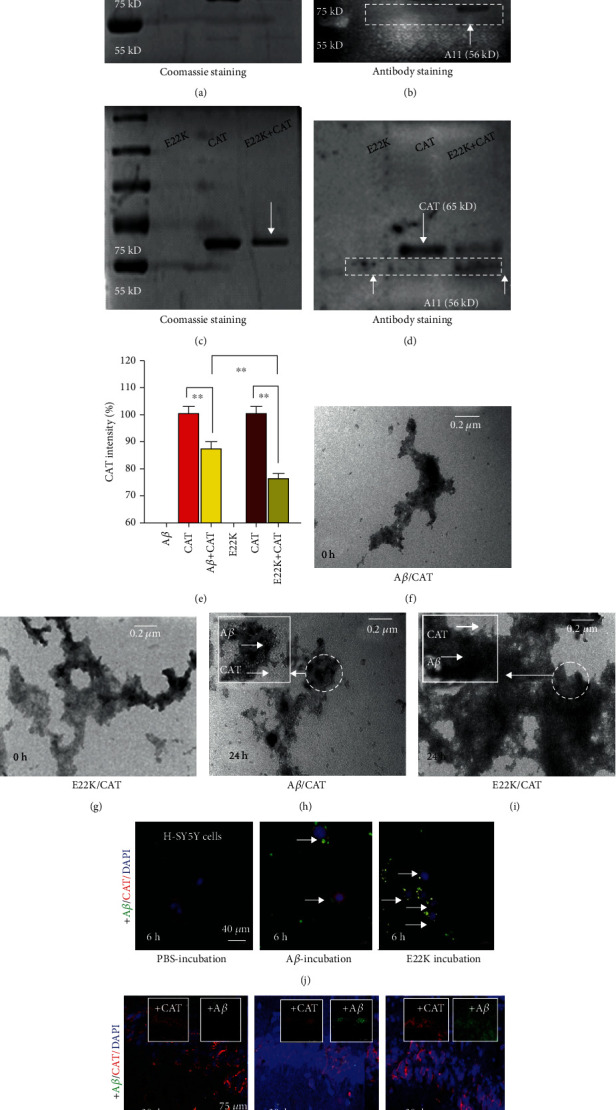
Identification of the complex structure of E22K/catalase and A*β*/catalase in vitro. (a–e) The binding strength between A*β* and catalase (0.05 mg/mL) and between E22K and catalase (0.05 mg/mL) as assessed by Coomassie Blue or by western blot (WT) analysis of catalase antibody and A11, respectively. The quantified WT band intensity of CAT after A*β* or E22K incubated with CAT (e). (f–i) A*β* (black) or E22K (black) induced the complex formation of A*β*/catalase or E22K/catalase (catalase, white balls) at 0 and 24 h (TEM). Bar: 0.2 *μ*m. (j) Colocalization of E22K (green) and catalase (red) after E22K or A*β* incubation for 6 hours in cultured human SY5Y cells. DAPI: nuclear stain, blue. (k) Colocalization of E22K (green) and catalase (red) after E22K or A*β* injection 30 days prior. All experiments were repeated in triplicate.

Next, we used the biochemical methods to identify the theoretical results of the molecular simulation. The sample solutions of A*β*, catalase, and an A*β*/catalase mixture were analyzed by western blotting. Using Coomassie Blue staining, we found catalase to be represented by a band at approximately 65 kD, while the 65 kD band (catalase) isolated from the mixed solution of A*β* and catalase was weaker than that isolated from the catalase alone (Figure 3(a)), suggesting that A*β* binding of catalase may slow the running speed of isolated glue, as compared with the bands for catalase or A*β* alone. Furthermore, we used a catalase antibody and the A11 antibody (which recognizes A*β* oligomers, for example, A*β*56 (56 kD)) to quantify the two proteins. Similarly, A*β* incubated with catalase markedly reduced the catalase band compared with catalase alone (Figure 3(b)). Catalase consists of four subunits, A*β* oligomers bound with the small subunit of catalase to form a coarser band than that of catalase or A*β* oligomers. This led to diminish the bands of the active four subunits of catalase and reduce its activity. Notably, E22K exhibited a marked reduction in the catalase band at 65 kD as assessed by both Coomassie Blue and antibody staining (Figures 3(c)–3(e)).

We used TEM to observe the molecular structure of E22K/catalase and A*β*/catalase complexes. In agreement with our western blot data, we found that E22K had a stronger ability to bind with catalase than A*β* (catalase: white dot; E22K or A*β*: black fibers) (Figures 3(f)–3(i)). Moreover, E22K incubation for 6 hours or injection 30 days prior indeed induced greater numbers of neurons with colocalization of E22K (green) and catalase (red) than wild-type A*β* treatment *in vitro* (Figure 3(j) and Figure S2) and *in vivo* (Figure 3(k) and Figure S3). Our data confirmed that there is a stronger binding between E22K and catalase than between A*β* and catalase.

### 3.4. H_2_O_2_ Combined with E22K Induced Stronger Aggregation Than with A*β* In Vitro

**Figure 4:**
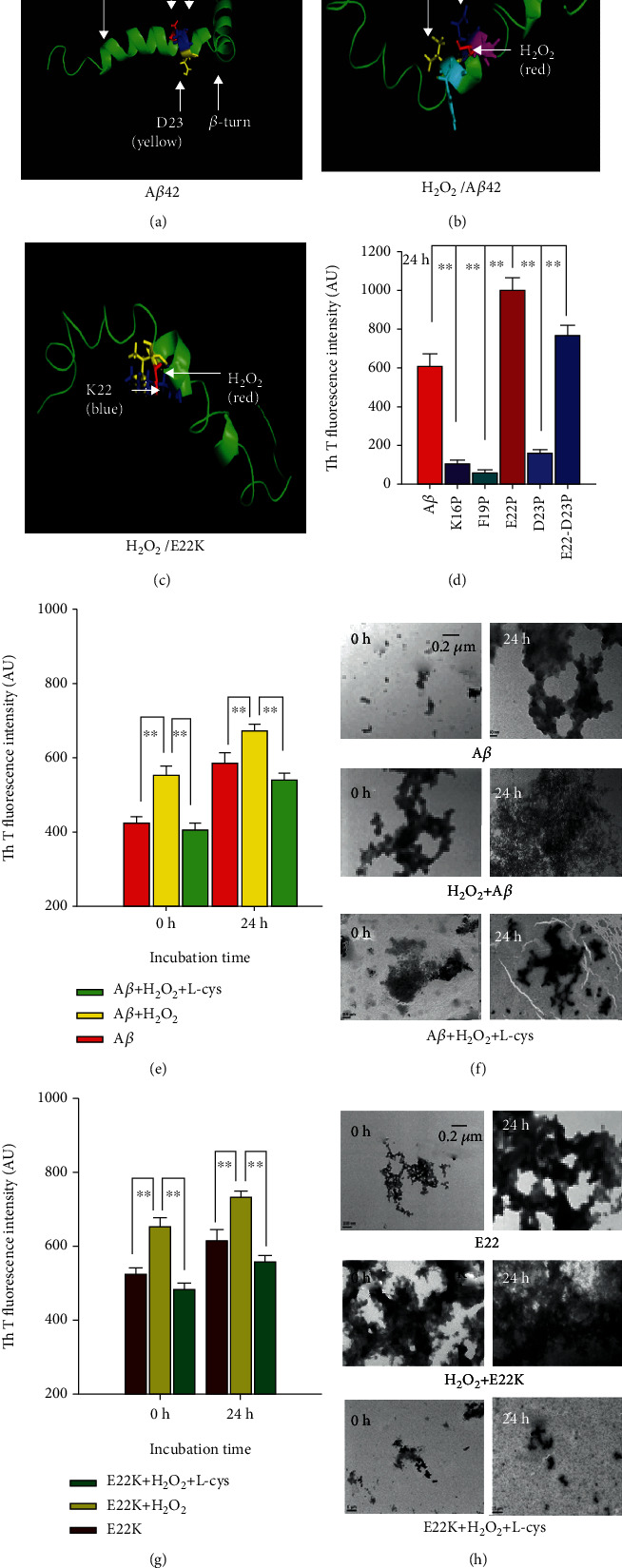
E22K induced stronger self-aggregation than A*β* in vitro. (a) The 21–23 residues in the *α*-helix structure of A*β* (PDB ID: 1IYT). (b) The theoretical simulated 3D crystal structure of H_2_O_2_ (red) binding with the E22 (blue) and D23 (purple) residues of A*β* (green). (c) The theoretical simulated 3D crystal structure of H_2_O_2_ (red) binding with the K22 (blue) residue of E22K (green). (d) The effects of a single mutation in K16P, F19P, E22P, and D23P, respectively, and a double mutation, E22D23P, on *β*-sheet formation as detected by Th T dye. (e–h) The effects of H_2_O_2_ and L-cys on *β*-sheet and fibril formation at 0 and 24 h as detected by Th T dye and TEM. Bar: 0.2 *μ*m. Experiments were repeated in triplicate. ^∗∗^*p* < 0.01. Data are means ± SEM.

Based on our above data, we speculated that H_2_O_2_ binding with K22 residue of E22K may induce stronger assembly than binding with A*β*. The results of molecular simulation showed that H_2_O_2_ (red) can bind with A*β* E22 (blue) and D23 (purple) residues through H-bonds (yellow dotted line) (Figures 4(a) and 4(b)). Notably, in the simulated H_2_O_2_/E22K 3D model, H_2_O_2_ (red) bound with the K22 (blue) residue of E22K (Figure 4(c)). Systematic replacement with proline (P) in peptides is a reliable and rapid method for predicting the secondary structure, especially in *β*-sheets and turns [27]. To test the above theoretical speculation, we used Th T dye to examine the changes in *β*-sheets of single point mutations including A*β* K16P, F19P, and D23P and a double mutation of E22P-D23P at 24 h. The results showed that K16P, F19P, and D23P induced *β*-sheet reduction while mutation of E22P led to stronger aggregation than the double mutation of E22P-D23P or wild-type A*β* (Figure 4(d)). Thus, the results of both the theoretical simulation and biochemical tests suggested that H_2_O_2_ binding with the K22 residue of E22K may induce stronger aggregation than H_2_O_2_ binding of A*β* at the E22 and D23 residues.

In fact, H_2_O_2_ significantly enhanced A*β* aggregation at 0 and 24 h compared with A*β* alone (0 h: 583.14 ± 30.18 vs. 421.68 ± 21.43; 24 h: 623.31 ± 36.72 vs. 551.02 ± 25.16; *n* = 6, *p* < 0.01); however, the H_2_O_2_ scavenger L-cys attenuated H_2_O_2_-induced A*β* aggregation *in vitro* (Figure 4(e)). This result was also confirmed by TEM (Figure 4(f)). Notably, H_2_O_2_ elicited a stronger assembly of E22K than A*β* at 0 and 24 h (0 h: 542.82 ± 24.30 vs. 421.68 ± 21.43; 24 h: 779.38 ± 49.73 vs. 583.14 ± 30.18, *n* = 6, *p* < 0.01; Figures 4(e)–4(h)); and L-cys weakened aggregation of E22K as detected by Th T dye and TEM (Figures 4(g) and 4(h)). The above data indicate that K22 is the critical target site for H_2_O_2_-promoting E22K aggregation, and L-cys attenuated H_2_O_2_-induced aggregation of E22K or A*β*.

### 3.5. Mutation at E22 Promoted More Severe Aggregation of E22K Than Wild-Type A*β*

**Figure 5:**
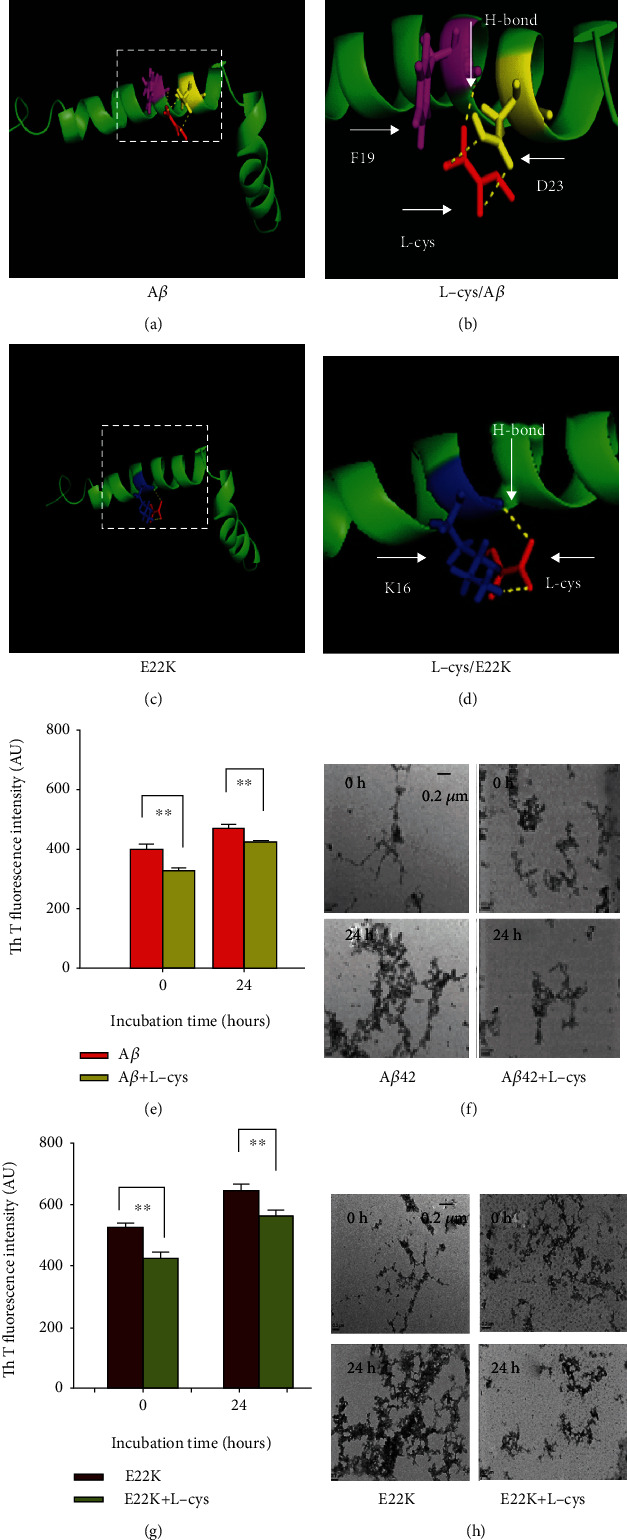
L-cysteine reduced the aggregation of E22K and A*β* in vitro. (a, b) The theoretical simulated 3D crystal structure of L-cys (red) binding with the F19 (purple) and D23 (yellow, full line) residues of A*β* by H-bonding (yellow dotted line). (c, d) The simulated 3D crystal structure of L-cys (red) binding with the K16 (blue) residue of E22K by H-bonding (yellow dotted line). (e–h) The effects of L-cys on *β*-sheet and fibril formation at 0 and 24 h as examined by Th T dye and TEM. Bar: 0.2 *μ*m. Experiments were repeated in triplicate. ^∗∗^*p* < 0.01. Data are the means ± SEM.

A previous study showed that L-cys reduced A*β* assembly *in vitro* [28], suggesting that L-cys most likely affected A*β* secondary structure. Our above data indicated that the K16P, F19P, or D23P mutations decreased A*β* aggregation (Figure 4(d)). We then examined whether the K16, F19, or D23 residues were the binding sites for L-cys and E22K or A*β*, respectively. Using the molecular simulation software, AutoDockTools-1.5.6, we found that L-cys (red) bound with the F19 (purple) and D23 (yellow full line) residues of A*β* through H-bonds (yellow dotted line) (Figures 5(a) and 5(b)). However, L-cys also bound with the K16 residue of E22K (Figures 5(c) and 5(d)). These data suggested that L-cys binding with K16 residue prevents E22K self-aggregation, and L-cys binding with F19 and D23 residues attenuates A*β* self-assembly.

Next, we used Th T dye and TEM to test the above results regarding theoretical simulation. The results showed that L-cys significantly reduced A*β β*-sheet formation, because Th T fluorescence intensity was markedly reduced at 0 h (410.28 ± 21.76 vs. 341.18 ± 18.23, *n* = 6, *p* < 0.01) and at 24 h (456.74 ± 31.98 vs. 420.46 ± 22.17, *n* = 6, *p* < 0.01; Figure 5(e)). Similarly, inhibition of A*β* fibril formation by L-cys was observed by TEM imaging (Figure 5(f)). We found that E22K significantly enhanced self-aggregation compared with A*β* at 0 and 24 h, respectively (0 h: 421.68 ± 21.43 vs. 583.14 ± 30.18, *n* = 6, *p* < 0.01; 24 h: 524.16 ± 12.34 vs. 623.31 ± 36.72, *n* = 6, *p* < 0.01; Figures 5(e) and 5(g)); however, L-cys reduced the formation of *β*-sheets and fibrils in E22K (Figures 5(g) and 5(h)). These data indicated that E22K promoted stronger self-aggregation than A*β*; while L-cys prevented self-aggregation of E22K and A*β*.

### 3.6. E22K Elicited Rapid Aggregation and Severe Neuronal Loss Compared with A*β*

**Figure 6:**
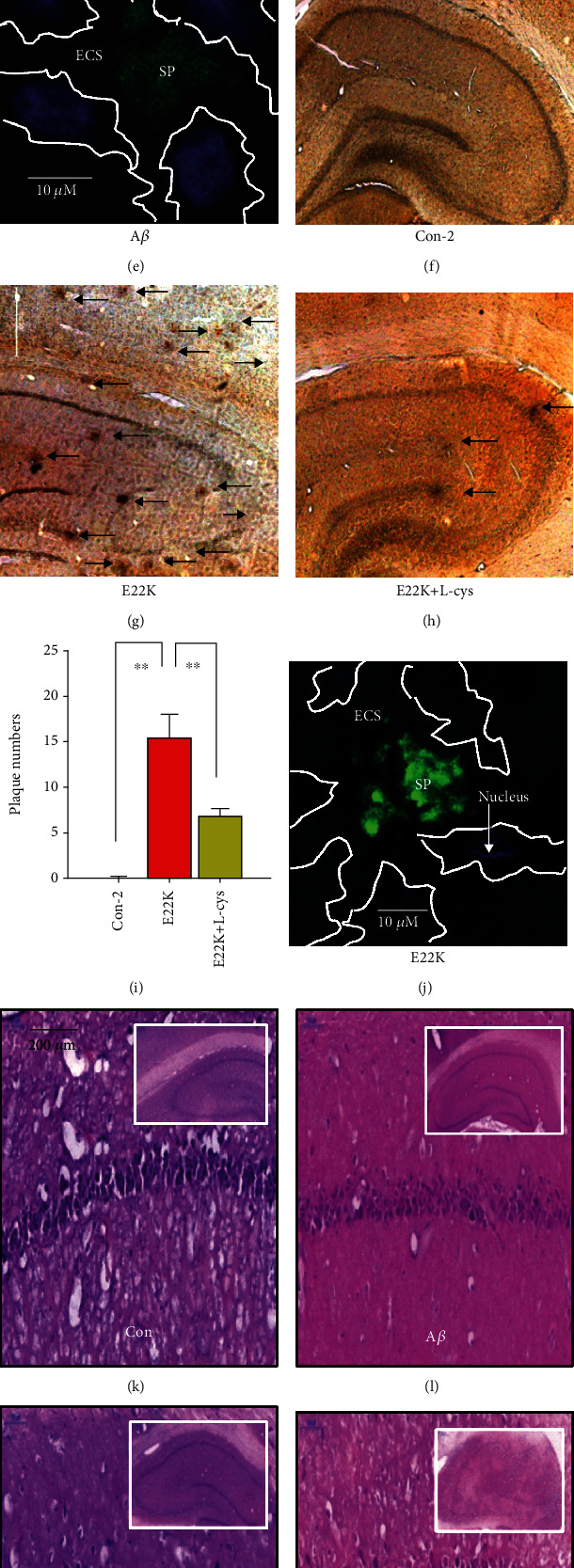
E22K injection induced more plaque deposition and neuronal loss compared with A*β* injection. (a–d) The effects of saline (Con), A*β*, and A*β* plus L-cys on the formation of senile plaques (SPs) in the hippocampi and cortices of rats quantified by immunochemistry using antibody-4G8. (e) ECS blockage by SPs, which were doubly stained with Th S dye for SPs (green) and DAPI dye for the nuclei of neurons (blue). ECS: extracellular space. (f–i) The effects of saline (Con), E22K, and E22K plus L-cys on SP formation. Scale bar, 500 *μ*m. *n* = 3–6 sections from 3–4 rats each. (j) ECS blockage by SPs, which were doubly stained with Th S dye for SPs (green) and DAPI dye for the nuclei of neurons (blue). (k–o) The effects of saline (Con), A*β*, A*β* plus L-cys, E22K, and E22K plus L-cys on the numbers of hippocampal neurons examined by H&E staining. Scale bar, 200 *μ*m. *n* = 3–6 sections from 3–4 rats each. (p) The statistical data of the relative numbers of hippocampal neurons in the above groups. ^∗∗^*p* < 0.01. Data are means ± SEM.

A*β*-injected Alzheimer's disease (AD) rats are a classical animal model used to mimic A*β*-induced dementia [29], and senile plaques can be observed on day 30 [30]. To examine plaque deposition and cellular toxicity of E22K *in vivo*, we injected E22K, E22K with L-cys, A*β*, and A*β* with L-cys, respectively, into the hippocampi of healthy adult male SD rats. At 30 days post injection, the brains were used for immunochemistry by using antibody-4G8, Th S staining, DAPI, and H&E. The results showed that A*β* injection resulted in a marked increase in the numbers of SP (A*β* group: 12.37 ± 0.46; Con group: 0.00 ± 0.00, *n* = 10, *p* < 0.01; Figures 6(a)–6(d)) and rapid SP deposition in the extracellular space (ECS) among hippocampal neurons compared with controls (Figure 6(e)); however, L-cys reduced SP in A*β*-injected SD rats (Figures 6(a)–6(c)). Surprisingly, E22K injection increased SP compared with A*β* injection (E22K group: 15.32 ± 2.52; A*β* group: 12.37 ± 0.46; *n* = 10, *p* < 0.01; Figures 6(f)–6(i)). More importantly, we observed more severe ECS blockage among the hippocampal neurons of E22K-injected rats, which was accompanied by severe neuronal death (fewer neuron nuclei stained by DAPI, blue) compared with A*β*-injected rats (Figures 6(e) and 6(j)). These results indicate that E22K causes more plaque deposition than did A*β in vivo*.

Next, we examined the relationship between ECS blockage and neuronal death. The results of H&E staining showed that spatial memory-related hippocampal neurons were significantly decreased in E22K-injected rats compared with A*β*-injected rats (Figures 6(k), 6(l), and 6(n)); however, L-cys reversed E22K- and A*β*-induced neuronal loss in the hippocampus (Figures 6(m), 6(o), and 6(p)). Thus, E22K induce more severe neurotoxicity than A*β*, while L-cys attenuated E22K toxicity.

### 3.7. E22K Induced More Severe Neuron Death and Memory Decline Than A*β*

**Figure 7:**
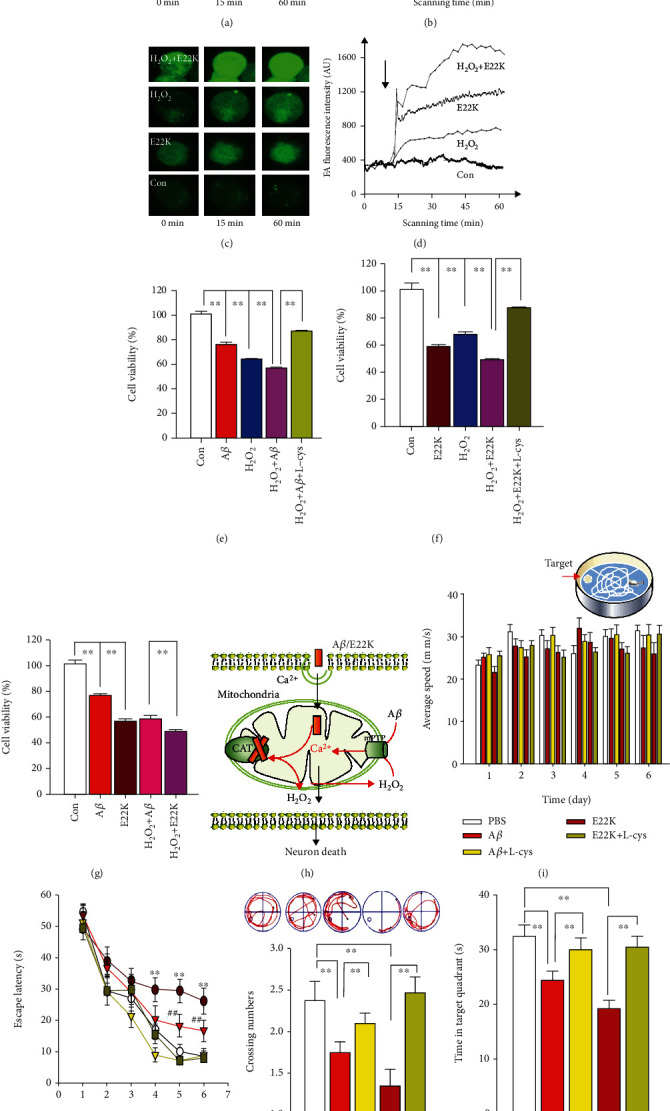
E22K induced stronger cell toxicity by eliciting intracellular Ca^2+^ influx [Ca^2+^]i in cultured N2a cells and more rapid memory decline than A*β* in SD rats. (a–d) The effects of saline (Con), A*β*, A*β* plus H_2_O_2_, E22K, and E22K plus H_2_O_2_ on intracellular Ca^2+^ levels by confocal microscopy (*n* = 3: number of independent cell culture preparations). (e–g) The effects of saline (Con), A*β*, A*β* plus L-cys, E22K, and E22K plus L-cys on cellular toxicity using a CCK-8 kit (*n* = 8: number of independent cell culture preparations). (h) The model of A*β* or E22K-induced neurotoxicity. Briefly, A*β* or E22K can enter into mitochondria and bind with catalase to induce H_2_O_2_ accumulation; in turn, H_2_O_2_ with A*β* or E22K acts to synergistically increase [Ca^2+^]i and to induce neuronal death (*n* = 6: number of independent cell culture preparations). ^∗∗^*p* < 0.01. Data are means ± SEM. (i) No difference in swimming speed among the five groups tested (saline (Con), A*β*, A*β* plus L-cys, E22K, and E22K plus L-cys) was observed in Morris water maze (MWM, above). (j) Escape latencies in the MWM. (k, l) Representative numbers of platform crossings (k, swimming trace, above) and (l) time spent in the target quadrant on day 7 (*n* = 10: number of rats). ^∗∗^*p* < 0.01. Data are means ± SEM.

To test that hippocampal neuronal death was due to E22K-induced neurotoxicity *in vitro*, we used confocal microscopy to scan the changes in the levels of [Ca^2+^]i using Ca^2+^-fluorescence probe-Fluo 4 in the cultured N2a cells treated with H_2_O_2_, E22K, H_2_O_2_ plus E22K, A*β*, and H_2_O_2_ plus A*β*, respectively. The results showed that H_2_O_2_, A*β*, or E22K significantly increased intracellular Ca^2+^ levels, respectively (Figures 7(a)–7(d)). Importantly, combined H_2_O_2_ and E22K treatment elicited a stronger [Ca^2+^]i than H_2_O_2_ plus A*β* (Figures 7(b) and 7(d)). These data suggested that a combination of H_2_O_2_ and E22K may induce more severe cellular toxicity than a combination of A*β* and H_2_O_2_.

Intracellular Ca^2+^ overload is known to cause cell apoptosis and/or death [17, 18]. To address whether E22K and H_2_O_2_ acted to synergistically accelerate cell death *in vitro*, we detected cellular viability using a CCK-8 kit after N2a cells were incubated with H_2_O_2_, E22K, H_2_O_2_ plus E22K, A*β*, and H_2_O_2_ plus A*β*, respectively. Our results showed that either A*β* or E22K coincubated with H_2_O_2_ significantly increased cellular toxicity compared with the respective treatments without H_2_O_2_; however, L-cys reduced the synergistic effect of H_2_O_2_ with both A*β* and E22K (Figures 7(e) and 7(f)). Notably, combined H_2_O_2_ and E22K treatment elicited stronger intracellular toxicity than a combination of A*β* and H_2_O_2_ (Figure 7(g)). In summary, E22K bound with catalase and induced H_2_O_2_; in turn, H_2_O_2_ and E22K acted synergistically to increase cellular toxicity (Figure 7(h)).

To address the most critical question whether E22K had more severe memory toxicity than A*β in vivo*, we examined the performance of memory behaviours in the MWM from day 23 to 30, after the five groups (*n* = 10, each group) were intrahippocampally injected with saline, A*β*, A*β* plus L-cys, E22K, and E22K plus L-cys, respectively. There was no difference in the swimming speed between the E22K-injected and A*β*-injected groups (Figure 7(i)). However, repeated measures two-way ANOVA revealed a difference in group: *F*_(4, 45)_ = 21.36, *p* < 0.001, time (day): *F*_(5,225)_ = 82.68, *p* < 0.001, and a group/time interaction: *F*_(20,225)_ = 5.21, *p* < 0.003. E22K-injected rats exhibited an earlier spatial memory deficit on day 4 (*F*_(2, 27)_ = 3.95, *p* = 0.004) and a more rapid memory decline on day 5 (*F*_(2, 27)_ = 7.39, *p* = 0.003) and day 6 (*F*_(2, 27)_ = 8.10, *p* = 0.001) than that of the A*β*-injected rats (Figure 7(j)). On day 7, E22K-injected rats had fewer numbers of platform crossings and shorter staying times in the target quadrant than A*β*-injected rats (6.02 ± 0.17 vs. 2.38 ± 0.09, *n* = 10, *p* < 0.01; Figures 7(k) and 7(l) and Figure S4). However, L-cys reversed E22K- and A*β*-induced recall impairments (Figures 7(j)−7(l)). These data indicated that E22K initiated early-onset memory decline while L-cys rescued memory function.

## 4. Discussion

In this study, we found that a single point mutation of E22K triggered early-onset memory decline in the early-onset familial AD model rats and AD patients. E22K binding of catalase led to higher levels of H_2_O_2_; in turn, H_2_O_2_ binding with the K22 residue of E22K promoted more aggregation, increased neuronal loss, and resulted in earlier memory decline than wild-type A*β*. Interestingly, L-cys contributed to the alleviation of symptoms in the E22K-injected model rat (Figure S5).

A large volume of evidence shows that A*β* can induce strong reactive oxygen species (ROS) generation, which accelerates cognitive decline in AD [13]. Further, molecular research showed that A*β* can bind with catalase and lead to H_2_O_2_ accumulation [24, 31, 32]. Notably, H_2_O_2_, an active component of ROS, has been found to accelerate A*β* aggregation *in vitro* [33]. Theoretically, the results of our molecular simulation showed that H_2_O_2_ could bind with the residues E22 and D23 of A*β*. Systematic replacement with proline (P) in peptides is a reliable and rapid method for predicting the secondary structure, especially in *β*-sheets and turns. The P residues are rarely present in *β*-sheet, whereas they are easily accommodated in a variety of turns [34]. In fact, the single point mutation of E22-to-P induced a marked increase in aggregation of A*β* while the point mutation of D23-to-P significantly decreased A*β* self-assembly. These data were in agreement with a previous report [34]. However, the double mutation of E22-to-p and D23-to-P in A*β* induced a higher level of aggregation than the wild-type A*β* but induced a lower degree of assembly than E22P. These data indicated that H_2_O_2_ binding with residues E22 and D23 of A*β* led to more severe aggregation than A*β* without H_2_O_2_ treatment. In this study, H_2_O_2_ induced more rapid assembly of E22K by binding to K22 residues.

However, our molecular simulation results showed that H_2_O_2_ does not bind 21G, 22G, 22Q, or 23N in early-onset familial AD associated with the A*β*42 mutations A21G (Flemish), E22G (Arctic), E22Q (Dutch), and D23N (Iowa) [35]. Interestingly, aging leads to a gradual accumulation of endogenous formaldehyde [23], and abnormally high levels of formaldehyde have been found in different types of dementia [36]. Formaldehyde can trigger A*β* aggregation *in vitro* [37]. Through theoretical simulation, we found that formaldehyde can bind A21G and E22G, but not E22Q and D23N. In fact, G (glycine) can react with formaldehyde spontaneously [38]. These data suggest that formaldehyde may accelerate the aggregation of A21G and E22G. Formaldehyde scavengers can rescue memory in the late-onset AD model of APP/PS1 mice [36], and they may potentially be used to treat early-onset familial AD. However, this speculation requires further investigation. These findings also indicate that early-onset dementia with different molecular pathogenesis mechanisms requires different precise drug therapy.

Whether the Italian E22K mutation can bind with catalase and then affect H_2_O_2_ levels is largely unclear. In this study, theoretically, E22K was more closely bound with residue Y357 of catalase than wild-type A*β*. Indeed, as expected, it induced stronger inhibition of catalase activity than A*β in vitro*. It also caused rapid accumulation of H_2_O_2_ in the blood and brains of E22K-injected rats than A*β*-injected rats. In turn, H_2_O_2_ binding with residue K22 of E22K led to more severe aggregation and neurotoxicity than H_2_O_2_ binding with wild-type A*β*. The single point mutation of E22-to-P of A*β* resulted in stronger self-assembly than wild-type A*β in vitro*. This result was consistent with a previous report [34], suggesting that the E22 residue of A*β* determined the degree of self-aggregation. Indeed, the side chains of lysine (K) residues are easily oxidized [39] and H_2_O_2_ is known to react with K residues [40] and may help to explain why H_2_O_2_ induced increased self-aggregation compared with A*β*. Furthermore, *in vivo* results showed that E22K injection led to higher numbers of SP, more severe neuronal loss, and earlier and more severe memory decline than A*β* injection. These data supported the notion that the single mutation of E22-to-K was more sensitive to oxidative stress than A*β*. In addition, catalase is a main enzyme for degrading H_2_O_2_. A*β* binding to catalase markedly inhibits CAT activity and promotes oxidative stress [31]. This leads to the accumulation of endogenous H_2_O_2_. Unexpectedly, in turn, excessive H_2_O_2_ enhances the generation of A*β* through JNK-dependent activation of gamma-secretase [41]. Hence, there is a H_2_O_2_-mediated vicious circle between catalase inactivation and A*β* production.

Another interesting finding was that L-cys reversed E22K-induced early-onset memory decline. A previous study proposed a theoretic mechanism by which E22K had a stronger aggregation potential than A*β in vitro*, because E22K oligomers and fibrils both displayed an antiparallel *β*-sheet structure, in comparison with the parallel *β*-sheet structure of wild-type A*β* [42]. Residues 21–23 of A*β* are considered to be critical for determining A*β* production, aggregation, and or clearance [43–46]. In this study, we found that mutation of K16-to-P significantly enhanced self-aggregation. This result was similar to previous reports [34] and indicated that the K16 residue determined the aggregation degree of A*β*. Concomitantly, using a theoretical molecular simulation, we found that L-cys bound with the K16 residue of E22K. In fact, the results of both Th T and TEM showed that L-cys significantly reduced self-assembly of E22K. L-cys bound with the F19 and D23 residues of wild-type A*β*. Through synthetic peptide mutations of A*β*, we found that single point mutations from F19-to-P or from D23-to-P markedly decreased self-aggregation. As expected, we found that L-cys reduced self-aggregation of A*β*. These data indicated that the binding sites between L-cys and A*β* determined the degree of self-aggregation. Another possible mechanism by which L-cys prevented H_2_O_2_-induced E22K aggregation was that L-cys directly scavenged H_2_O_2_. N-acetyl-L-cysteine (NAC) is synthesized by L-cys and is known to be an H_2_O_2_ scavenger [47]; furthermore, it is also known that L-cys reacts with H_2_O_2_ [25]. Thus, L-cys or its precursor NAC is likely a potential therapeutic method to treat early-onset Italian familial AD.

## 5. Conclusion

Only single point mutation from E22-to-K of A*β* was enough to promote the outbreak of early-onset Italian familial AD in a rat model and patients. L-cys may contribute to the treatment of this neurodegenerative disease.

## Data Availability

The data used to support the findings of this study are available from the corresponding author upon request.

## Supplementary Materials

Supplementary Materials Figure S1: the synthetic wild-type A*β* and E22K mutant identified by liquid chromatography-tandem mass spectrometry (LC-MS/MS). Figure S2: the localization of A*β* with catalase (CAT) in the cultured human SY5Y cells (H-SY5Y) after PBS, A*β*42, or E22K incubation for 6 hours. Figure S3: the localization of A*β* with catalase (CAT) in the hippocampus of these SD rats injected with PBS, A*β*, or E22K on day 30. Figure S4: time in different quadrants of the five groups of mice in the Morris water maze on day 7. The adjacent left (AdjL), training (Tra), adjacent right (AdjR), and opposite (Opp) quadrants. Figure S5: the model of early-onset Italian familial Alzheimer's disease. Briefly, E22K induces higher levels of H_2_O_2_ by rapidly inhibiting catalase activity than A*β*. Excessive H_2_O_2_ elicits severe E22K assemblies and neuron loss and then results in the early-onset AD. However, L-cysteine (L-cys) rescues memory functions. AD: Alzheimer's disease; SP: senile plaques.
