# Supplementary material for: Single Point Mutation from E22-to-K in Aβ Initiates Early-Onset Alzheimer's Disease by Binding with Catalase
Source: Oxid Med Cell Longev. 2020 Dec 24;2020:4981204. doi: 10.1155/2020/4981204 (PMC7775154; doi:10.1155/2020/4981204)
Supplement: Supplementary Materials — Figure S1: the synthetic wild-type Aβ and E22K mutant identified by liquid chromatography-tandem mass spectrometry (LC-MS/MS). Figure S2: the localization of Aβ with catalase (CAT) in the cultured human SY5Y cells (H-SY5Y) after PBS, Aβ42, or E22K incubation for 6 hours. Figure S3: the localization of Aβ with catalase (CAT) in the hippocampus of these SD rats injected with PBS, Aβ, or E22K on day 30. Figure S4: time in different quadrants of the five groups of mice in the Morris water maze on day 7. The adjacent left (AdjL), training (Tra), adjacent right (AdjR), and opposite (Opp) quadrants. Figure S5: the model of early-onset Italian familial Alzheimer's disease. Briefly, E22K induces higher levels of H2O2 by rapidly inhibiting catalase activity than Aβ. Excessive H2O2 elicits severe E22K assemblies and neuron loss and then results in the early-onset AD. However, L-cysteine (L-cys) rescues memory functions. AD: Alzheimer's disease; SP: senile plaques. [file 4981204.f1.pdf]

Supplementary Figure 1

A

	1 10	20	30	40	42
**Aβ1-42 (wild-type)**	**DAEFRHDSGY**	**EVHHQKLVFF**	**AEDVGSNKGA**	**IIGLMVGGVVIA**	**IA**
**E22K (Itanian)**	**DAEFRHDSGY**	**EVHHQKLVFF**	**AKDVGSNKGA**	**IIGLMVGGVVIA**	**IA**

B

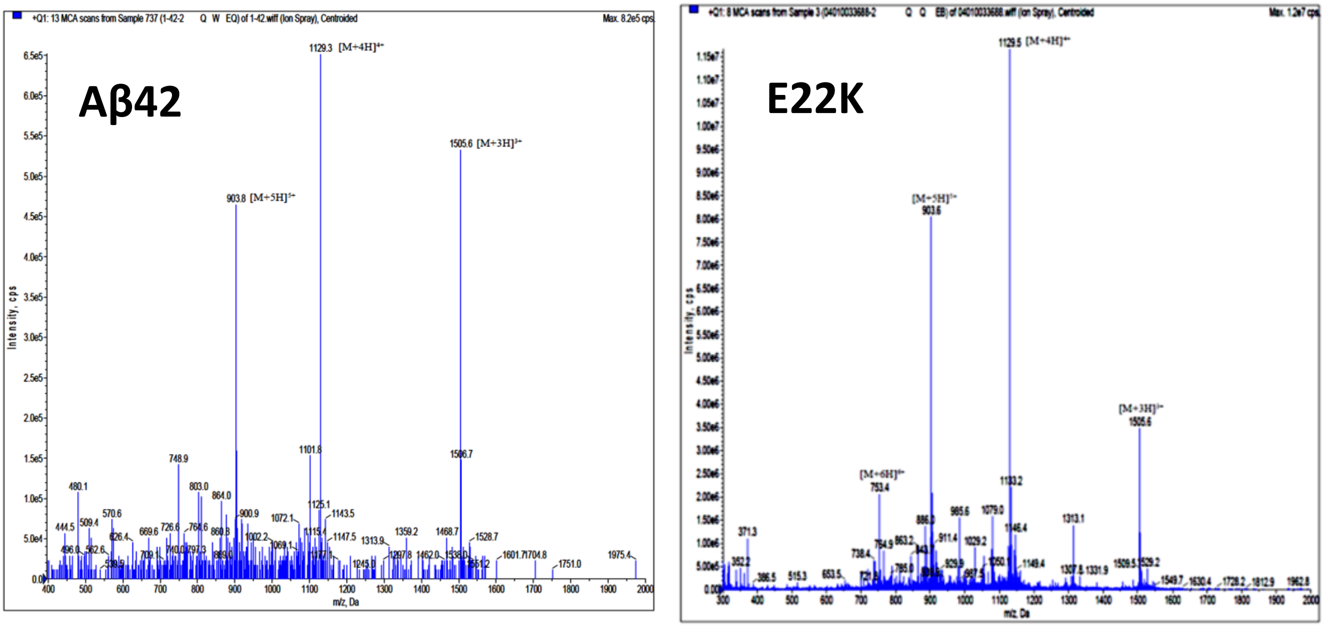

Figure S1. The synthetic wild-type Aβ and E22K mutant identified by liquid chromatography-tandem mass spectrometry (LC-MS/MS).

Supplementary Figure 2

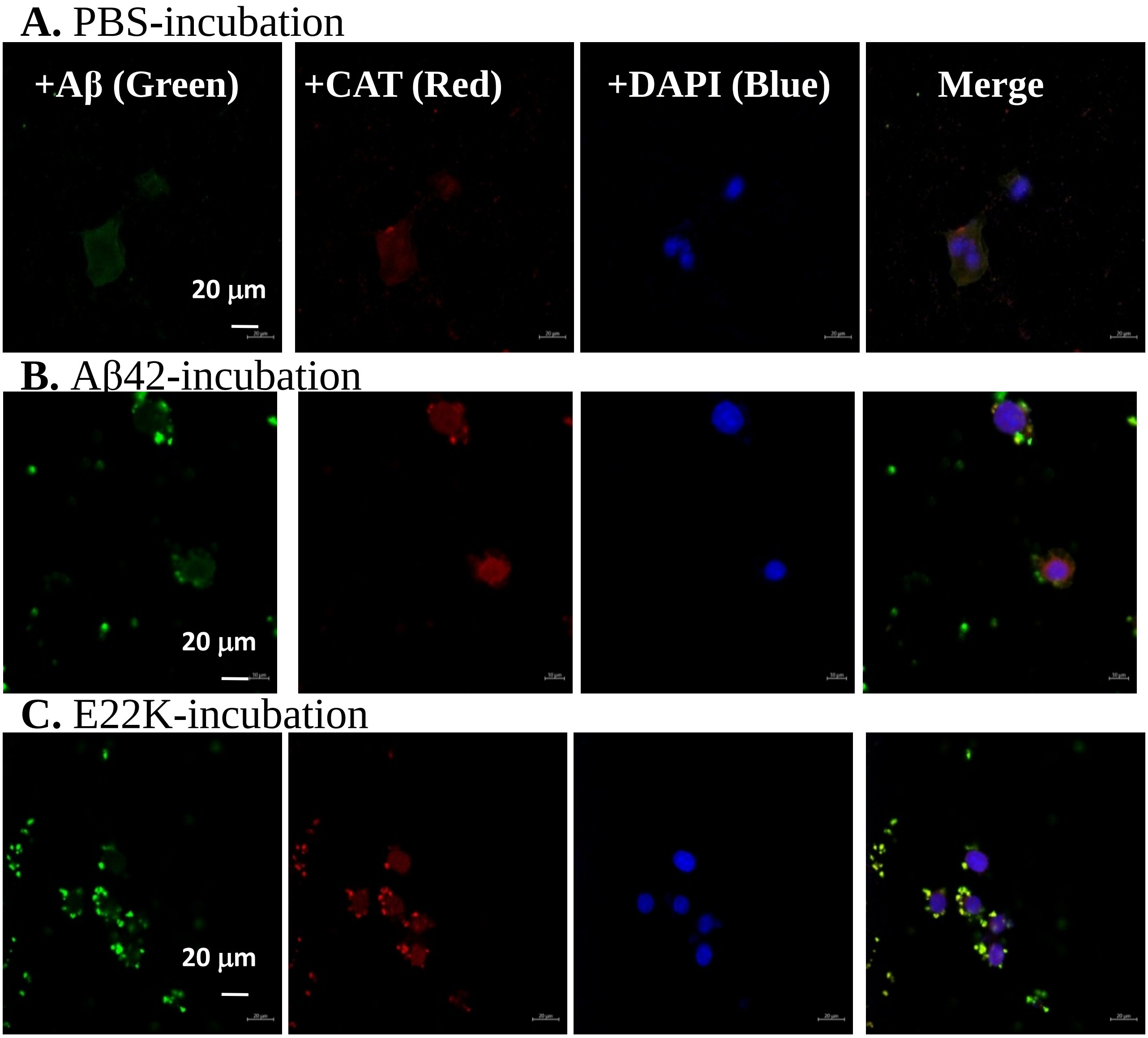

Fig. S2. The localization of Aβ with catalase (CAT) in the cultured human SY5Y cells (H-SY5Y) after PBS, Aβ42, or E22K incubation 6 hours.

Supplementary Figure 3

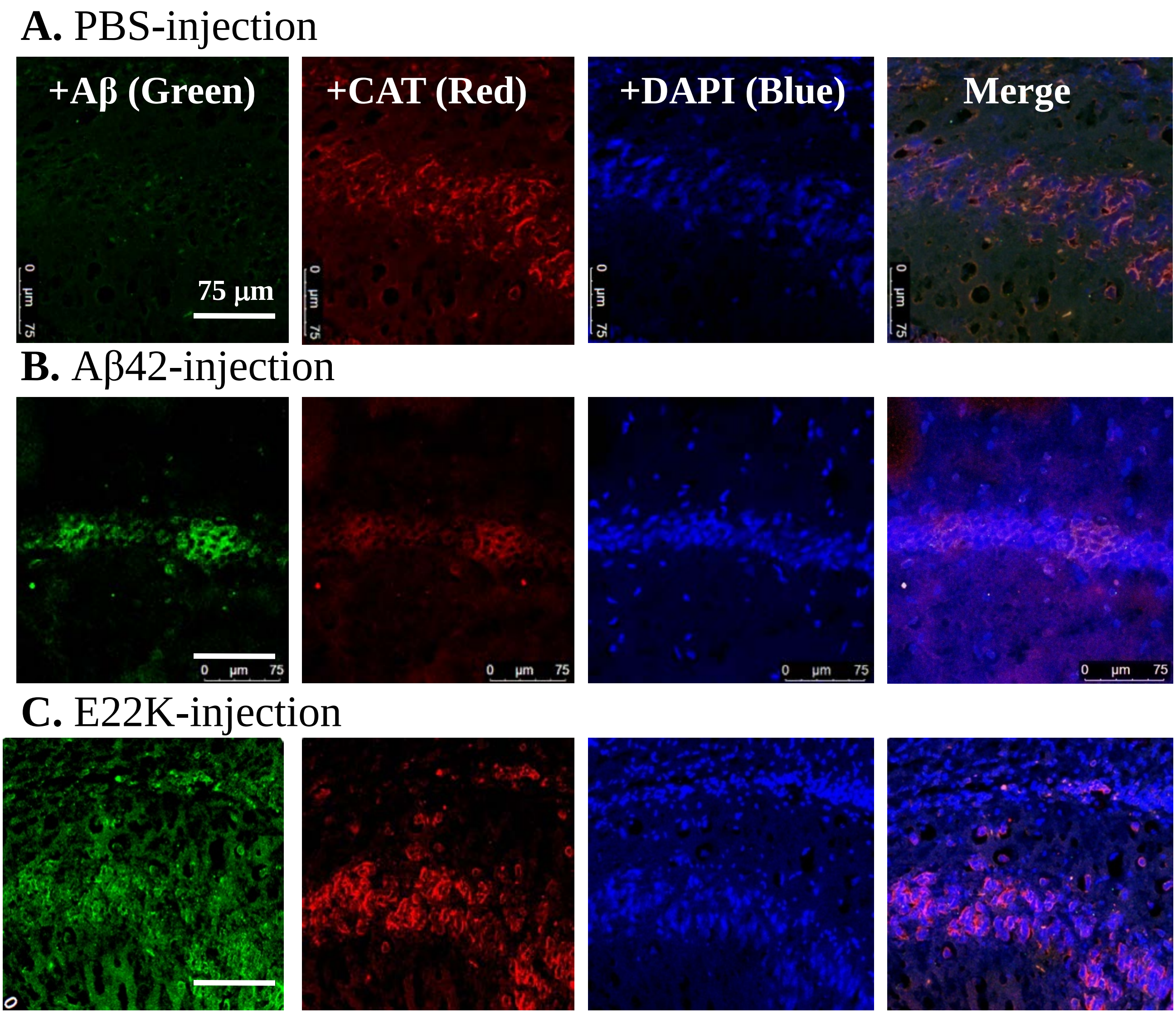

Fig. S3. The localization of Aβ with catalase(CAT) in the hippocampus of these SD rats injected with PBS, Aβ, or E22K on day 30.

Supplementary Figure 4

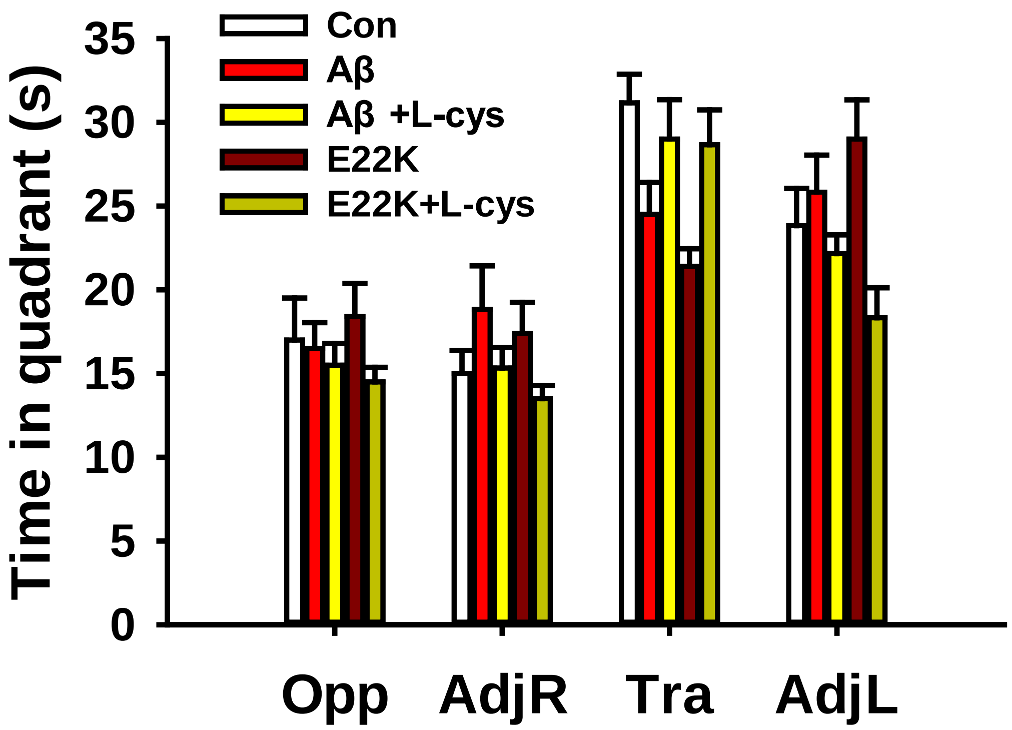

Fig. S4. Time in different quadrants of the five groups of mice in the Morris water maze on day 7. The adjacent left (AdjL), training (Tra), adjacent right (AdjR), and opposite (Opp) quadrants.

Supplementary Figure 5

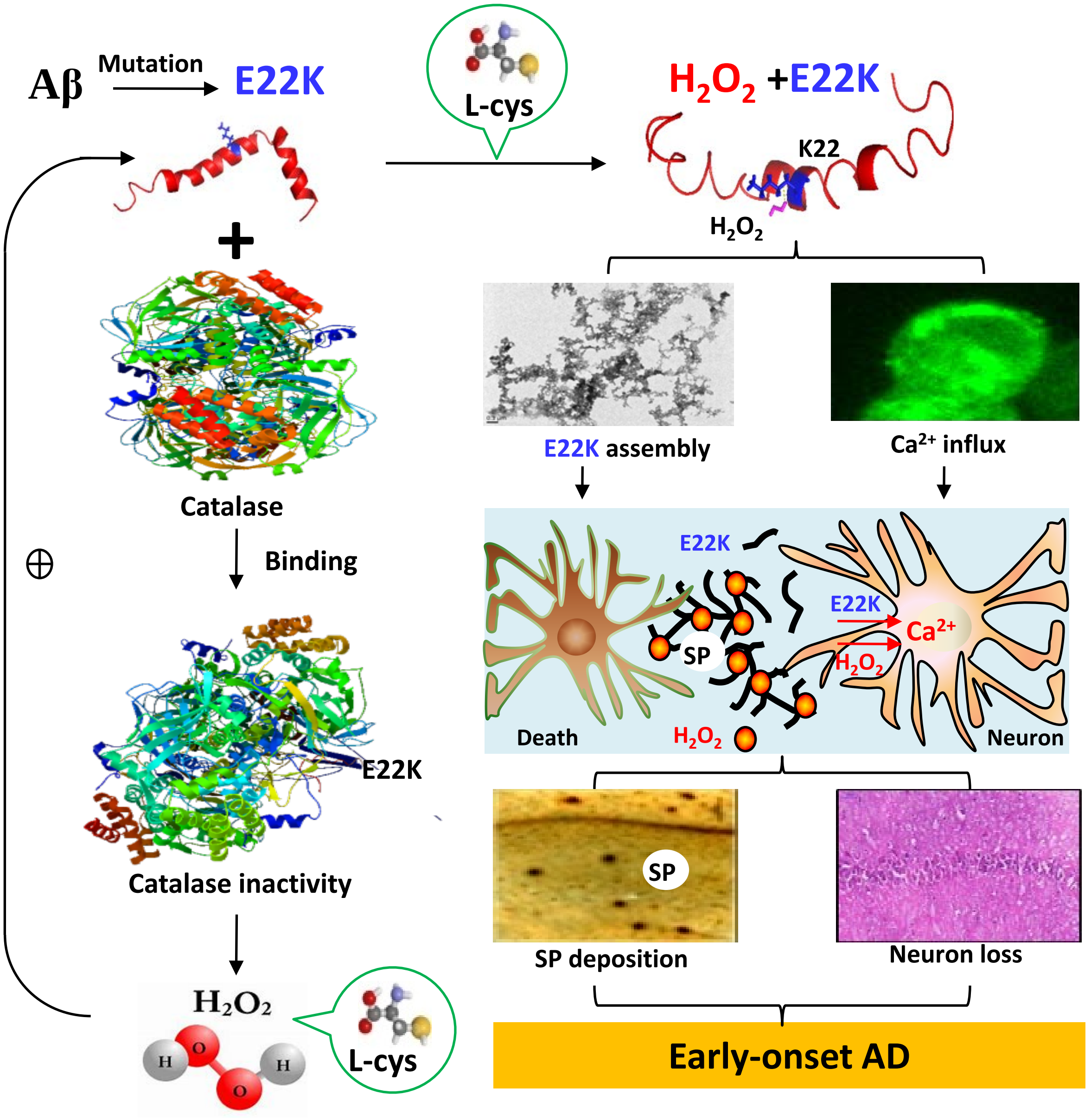

Fig. S5. The model of early-onset Italian familiar Alzheimer's disease. Briefly, E22K induces higher levels of H_2O_2 generation by rapidly inhibiting catalase activity than Aβ. Excessive H_2O_2 elicits severe E22K assemblies and neuron loss, and then results in the early-onset AD. However, L-cysteine (L-cys) rescues memory functions. AD: Alzheimer's disease, SP: senile plaques.
